# Monkey Meltdowns: Do Tantrums Influence Maternal Investment in Bearded Capuchin Monkeys?

**DOI:** 10.1002/dev.70157

**Published:** 2026-05-08

**Authors:** Mábia B. Cera, Guilbert Araujo, Helena F. Lima, Mateus Oka, Vinícius Andrietta, Patrícia Izar

**Affiliations:** ^1^ Institute of Psychology University of São Paulo São Paulo Brazil; ^2^ Institute of Environmental, Chemistry, and Pharmaceutical Sciences Federal University of São Paulo São Paulo Brazil; ^3^ Institute of Philosophy and Human Sciences State University of Campinas São Paulo Brazil; ^4^ Institute of Biosciences São Paulo State University São Vicente Brazil

**Keywords:** infant development, mother–infant interaction, parental investment, parent–offspring conflict, *Sapajus*, weaning

## Abstract

Tantrums are widely discussed in human development literature and are typically defined as displays of frustration or anger. In behavioral ecology, they are hypothesized to function as a strategy for securing greater maternal investment. Infant primates often exhibit tantrums when their attempts to obtain maternal care are rejected. We investigated whether these displays influence maternal investment in a wild population of capuchin monkeys (*Sapajus libidinosus*) at Fazenda Boa Vista, Brazil. We analyzed 213 h of footage of 12 infants filmed over their first 18 months of life using focal animal sampling and identified 550 infant solicitation events for nursing and transportation. Maternal rejection was rare (*N* = 47) but increased significantly with age. Infants displayed tantrums after most rejections (*N* = 31), but their occurrence did not vary significantly with age and had no significant effect on the likelihood of obtaining maternal care. Infant solicitations declined significantly with age, suggesting that maternal investment decreases as infants’ behavior changes. Our findings suggest that tantrums may reflect the challenges infants face during the transition to independence associated with weaning rather than an effective strategy for securing additional maternal care. By not reinforcing tantrums, mothers may facilitate infants’ gradual adjustment and shift toward greater independence.

## 1 Introduction

Temper tantrums are a common and well‐studied behavior in human children (e.g., Bhatia et al. 1990; Beauchamp‐Châtel et al. 2019; Prutipaisan et al. 2025). These episodes involve intense motor and vocal displays, such as kicking, stamping, falling to the floor, screaming, crying, and whining (Potegal et al. 1996; Green et al. 2011). They are considered to be an expression of extreme frustration or anger (Daniels et al. 2012), particularly in young children who are still developing emotional regulation (Österman and Björkqvist 2010). Interestingly, tantrum behaviors are not exclusive to humans. Similar displays have been documented in nonhuman primates (hereafter, primates), especially in the context of maternal rejection during weaning (Maestripieri 2002). Like human children, primate infants may scream, hurl themselves to the ground, and intensify contact‐seeking efforts toward their mothers when rejected (Barrett and Henzi 2000).

In behavioral ecology, tantrums are often framed within the parent–offspring conflict theory (Trivers 1974). According to this framework, while parents aim to optimize their overall reproductive success by regulating investment across offspring, each infant employs behavioral strategies to maximize its own fitness. Intense begging and tantrums may therefore function to exaggerate need and secure greater care. However, parent–offspring dynamic is not determined solely by genetic factors. For instance, C. A. Hinde et al. (2009) found that nestling begging intensity in birds was associated with ecological conditions experienced by parents during nesting and egg‐laying. Similarly, Caro et al. (2016) showed that in predictable, resource‐rich environments, avian parents were more responsive to chicks that beg more intensely. In contrast, under poor environmental conditions, parents relied more on cues of quality (e.g., colorful mouths, larger bodies) rather than need and were less responsive to begging intensity.

Bird studies commonly explore these proximate mechanisms underlying parent–offspring dynamics, particularly the relationship between parental provisioning and nestling vocalizations (e.g., Stamps et al. 1985; C. A. Hinde et al. 2009; Caro et al. 2016). In contrast, little attention has been given to whether tantrums are effective in eliciting increased care in mammals. In humans, Potegal and Davidson (1997) found that more intense and distressed tantrums increased the likelihood of post‐tantrum affiliation. In primates, Berman et al. (1993) reported that rhesus macaques (*Macaca mulatta*) infants who displayed more tantrums experienced longer suckling bouts and higher rates of nipple contact. Conversely, Devinney et al. (2001) found that infants showing greater distress (e.g., screaming) were approached less frequently by their mothers.

Ecological conditions may partly explain these inconsistencies. Hauser and Fairbanks (1988) found that, in vervet monkeys (*Chlorocebus pygerythrus*), when high‐quality food was available, mothers had shorter interbirth intervals and reduced investment in current offspring, increasing behavioral conflict. In low‐quality environments, longer interbirth intervals allowed for more prolonged care. Dezeure et al. (2021) reported that baboon infants born at suboptimal times (i.e., facing the dry season at the end of weaning) showed more tantrums after rejection, and mothers increased their investment during the dry season. However, they did so independent of the offspring's age, not only in response to the tantrum displays.

The effects of tantrums on maternal investment have yielded mixed results and, although ecological conditions may account for part of the variation, this phenomenon remains largely underexplored, particularly in Platyrrhini species. In some of these species, maternal rejection is reported to be infrequent and rarely aggressive (e.g., *Ateles paniscus*: Carvalho and Otta 1998; *Sapajus* spp.: Verderane and Izar 2019; *Alouatta caraya*: Pavé et al. 2010, 2015; *Ateles geoffroyi*: Arbaiza‐Bayona et al. 2022). However, to our knowledge, none of these studies have investigated the role of tantrums in shaping maternal investment.

This study aimed to investigate how infant reactions to maternal rejections influence maternal investment in a population of bearded capuchin monkeys (*Sapajus libidinosus*). Capuchins exhibit prolonged maternal care characterized by intimate interactions (Fragaszy et al. 1991). Infancy lasts approximately 18 months, with nursing, transportation, and physical proximity gradually decreasing over this period (Verderane and Izar 2019). Behavioral conflicts during weaning have been documented in semi‐free‐ranging groups, with reports of infants responding to maternal rejection with tantrums and behavioral regression to earlier developmental stages (Verderane and Izar 2019).

If tantrums effectively influence maternal investment, we predict an increase in maternal rejection during weaning, followed by a rise in tantrum occurrence. In turn, this should lead to an increase in maternal responsiveness (i.e., infants successfully regain nipple access or are carried after a tantrum). We also expect an overall increase in both infant solicitation rates and maternal responsiveness, as tantrums are reinforced. This increase should be temporary, with both maternal investment and tantrum occurrence declining subsequently.

## 2 Methods

### 2.1 . Study Area and Subjects

Data were collected at Fazenda Boa Vista (FBV), located in Gilbués, northeastern Brazil (9°39ʹ36ʺ S, 45°25ʹ10ʺ W). FBV is situated in the Cerrado–Caatinga ecotone, and the vegetation is characterized by trees, shrubs, and palms (Visalberghi et al. 2007). It has two seasons defined by rainfall patterns: the wet season, from October to April, and the dry season, from May to September (Spagnoletti et al. 2012). Food availability remains high throughout the year (Mendonça‐Furtado et al. 2014). Potential primate predators in the area include carnivores such as *Puma concolor* and *Eyra barbara*, birds of prey like *Geranoaetus melanoleucus*, *Caracara plancus*, and *Herpetotheres cachinnans*, and the constrictor snake *Boa constrictor*.

We collected data from one wild group of bearded capuchin monkeys (*S. libidinosus*). They form a multimale, multifemale group, with philopatric females and a linear dominance hierarchy (Verderane et al. 2013; Mendonça‐Furtado et al. 2014; Izar et al. 2021). The population has been monitored by researchers since 2006 (Izar et al. 2012), and all subjects were habituated and individually recognized.

### 2.2 . Data Collection

The infants were filmed by the field collaborators Marcos Fonseca de Oliveira, Claudio Fonseca, and Arizomar de Oliveira using focal animal sampling (Altmann 1974). Each focal monkey was observed once a week for an entire day, from dawn to dusk, and filmed whenever visible. The infants were filmed from birth to the 36th month of life.

For this research, we selected a subset of videos from a broader database of the Laboratory of Ethology, Development, and Social Interaction at the Institute of Psychology of the University of São Paulo, owned and managed by P.I. The videos are also used in other research projects and are available upon reasonable request at *ledsapajus.ip@usp.br*. The subset analyzed was recorded between 2014 and 2017. During this period, the group size ranged from 18 to 29 individuals (Araujo et al. 2024). We selected 12 mother–infant dyads based on the regularity and availability of footage for each dyad. We analyzed footage of the focal infants at 10 timepoints: Months 2, 4, 6, 8, 9, 10, 12, 14, 16, and 18. This time frame (from birth to the 18th month of life) encompasses the infancy period for *Sapajus* spp. (Fragaszy et al. 2004; Verderane and Izar 2019). Analyzing every other month allowed us to cover a longer period of time while maximizing video analysis effort. We included the ninth month in our sampling based on evidence from Verderane and Izar (2019), showing that capuchin infants in a semi‐free‐ranging group reacted more intensely to maternal rejections during this period. All infants in this study were cared for by their biological mothers. Table 1 provides information on infants’ sex, birth, maternal parity, total sampling hours, and the number of episodes analyzed for each subject.

**TABLE 1:** Information of focal infants and mothers.

Subject	Affiliation	Infant sex	Birth (month/year)	Maternal parity	Sampling hours / number of episodes
Duca	Dita	Female	10/2014	Multiparous	19:01:29 / 85
Peteca	Piaçava	Female	11/2014	Multiparous	26:35:08 / 101
Olívia	Doree	Female	01/2015	Multiparous	21:48:29 / 47
Cacau	Chuchu	Male	03/2015	Multiparous	34:14:02 / 64
Dançarina	Dita	Female	02/2016	Multiparous	18:38:53 / 30
Hortelã	Chani	Male	11/2016	Primiparous	12:56:35 / 28
Oliveira	Doree	Male	11/2016	Multiparous	10:22:40 / 26
Michele	Pamonha	Female	12/2016	Multiparous	9:51:43 / 32
Dourado	Dita	Male	03/2016	Multiparous	15:31:19 / 20
Acerola	Paçoca	Female	03/2017	Multiparous	15:00:30 / 47
Pimenta	Piaçava	Female	05/2017	Multiparous	15:08:45 / 42
Caititu	Chuchu	Male	06/2017	Multiparous	13:58:49 / 28

### 2.3 . Video Analysis

M.B.C., H.F.L., M.O., and V.A. thoroughly screened all footage available for the focal infants to identify recordings in which infants solicited maternal care, such as nursing or carrying. Videos with low visibility were excluded, and only episodes showing clear mother–infant interactions from their onset were selected for analysis. We screened 5353 videos, totaling 213 h and 16 min of footage, and identified 550 episodes suitable for analysis.

M.B.C. analyzed all selected episodes in slow motion using BORIS (Behavioral Observation Research Interactive Software, version 7.10.7; Friard and Gamba 2016). Behavioral scoring began at the moment a solicitation of maternal care was identified. When the mother accepted the solicitation without signs of resistance or rejection (i.e., without behaviors such as moving away, hitting, or biting the infant), nursing and carrying events were recorded from their onset to their conclusion. If the footage was interrupted before the interaction concluded, the termination of the event was logged at the same moment the video ended. In episodes where the mother rejected the solicitation or interrupted the interaction, all behaviors performed by both the infant and the mother were recorded from the beginning of the episode until the end of the video recording. Two examples of this type of episode are provided in the Supporting Information (Video S1). A 5‐s latency between episodes of the same behavior was established (Verderane et al. 2019). Hence, if an infant stopped nursing for at least 5 s and then resumed, a new episode was logged. The ethogram (Supporting Information; Table S1) used for video analysis was developed based on preliminary observations conducted during the video screening and informed by previously published definitions (Gomendio 1991; Pavé et al. 2010, 2015; Verderane and Izar 2019).

After behavioral scoring, we categorized episode outcomes. We classified infant tantrums into two intensity levels: (a) mild, defined as cases in which, following maternal rejection, the infant displayed a combination of behaviors, such as approaching or moving away from the mother, vocalizing, and/or self‐scratching, and (b) intense, defined as cases in which these behaviors were accompanied by those described under the “intense distress” category in the ethogram (Table S1). For each tantrum episode, we registered the duration, in seconds, of each intensity level. Additionally, we also classified all episodes according to whether the infant succeeded in obtaining maternal care (coded as a binary variable, 0 = failure and 1 = success) and whether a tantrum occurred (coded as a binary variable, 0 = no tantrum and 1 = with tantrum). Maternal behavior was used solely to determine whether the mother rejected the infant solicitation.

Twenty‐five percent of the videos were independently scored by M.O., and interobserver reliability was assessed using Kendall's concordance coefficient (*W*). Only behaviors with agreement values of *W* ≥ 0.7 were retained for analysis. For this reason, the behaviors “hold tail” (*W* = 0.5), “hit” (*W* = 0.5), “remove infant from the nipple” (*W* = 0.375), and “remove infant's hand from the nipple” (*W* = 0.417) were excluded. Their exclusion did not compromise the analyses, as these behaviors consistently co‐occurred with other retained behaviors that allowed reliable episode classification. For example, when mothers removed infants from the nipple, they simultaneously displayed behaviors such as moving away, pushing, or holding the infant, all of which were retained in the dataset.

Within the context of this study, the behaviors allotransportation and socialize/conflict with another individual were recorded only when they occurred following a maternal rejection. Because these behaviors were rarely observed, they were not included in subsequent analyses.

### 2.4 . Statistical Analysis

We ran a generalized linear mixed model (GLMM) to quantify the variation in solicitation rate across the different ages (Model 1) using a negative binomial distribution due to significant overdispersion in the data (Cameron & Trivedi test: *p* < 0.001). In this model, we included the observation time as an offset to account for the different total observation time in each month. We ran three binomial GLMMs to test the effect of age on the probability of infant success in obtaining maternal care (Model 2), maternal rejection (Model 3), and tantrum occurrence (Model 4). In these models, we used the “cbind” function to account for the different proportion of episodes in each month. We also ran a binomial GLMM to test the effect of mild and intense tantrums on the probability of infant success in obtaining maternal care (Model 5).

In all the models, we included infant identity as a random effect to control for the repeated observations. We also included mother identity and infant sex as fixed effects, because mothers could have individual variations in their maternal style (Altmann 1980) and infant sex is a variable that can affect mother–infant dynamics in primates (Maestripieri 2018). To select the minimum adequate models, we performed ANOVA tests comparing full to simplified models.

All analyses were conducted in R v. 4.5.0. To run the GLMMs, we used the “glmer.nb” and “glmer” functions in the lme4 package (Bates et al. 2015). To diagnose the presence of overdispersion, we used the “overdisp” function (Souza et al. 2022). To select the minimum adequate models, we used the “anova” function on the *car* package (Fox and Weisberg 2019). We verified the distribution of residuals with the “residuals” function from stats and diagnostic plots (residuals vs. fitted values, histograms, and *Q*–*Q* plots). For binomial GLMM, we assessed model performance through ROC curves and AUC values using the pROC package (Robin et al. 2011). Detailed R script and report for all analyses are available in the Supporting Information (Statistical Report S1).

### 2.5 . Ethical Note

This research complied with protocols approved by the Animal Research Ethics Committee of the Institute of Psychology of the University of São Paulo (CEUA/IP 6870180216) and Brazilian law under the authorization from ICMBio (#47501‐9), and adhered to the American Society of Primatologists (ASP) Principles for the Ethical Treatment of Non‐Human Primates.

## 3 Results

**FIGURE 1:**
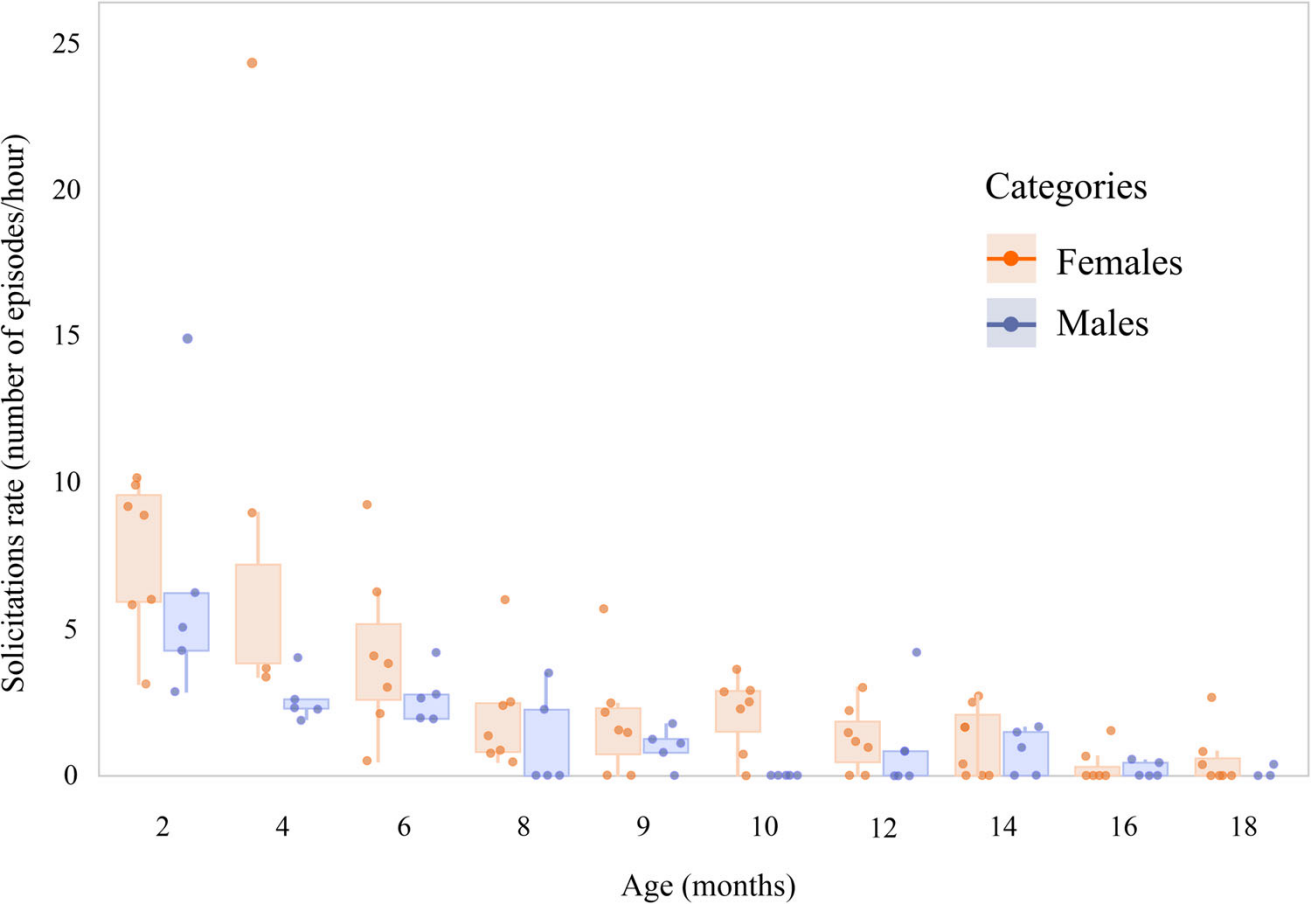
Rate of maternal care solicitation according to infant age and sex. Data are from 12 infants from a wild population of *Sapajus libidinosus* at Fazenda Boa Vista, Brazil. Infants are highlighted as circles.

**FIGURE 2:**
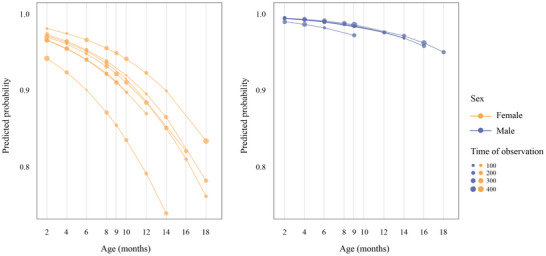
Predicted probability of infant success in obtaining maternal care per subject according to infant age. (a) Data from seven females. (b) Data from five males. Data are from a wild population of *Sapajus libidinosus* at Fazenda Boa Vista, Brazil. For both plots, the size of the circles is proportional to the total time of observation.

Solicitations by infants reduced significantly with age (Model 1: estimate ± *SE* = −0.18 ± 0.02; *p* < 0.001). Males solicited investment significantly less than females (Model 1: estimate ± *SE* = −0.54 ± 0.22; *p* = 0.02), and maternal care was still present by Month 18, indicating that some infants were not yet fully weaned (Figure 1). The probability of obtaining investment also decreased significantly with age (Model 2: estimate ± *SE* = −0.15 ± 0.05; *p* < 0.001). Notably, this reduction was less pronounced for males than for females (Model 2: estimate ± *SE* = 1.61 ± 0.82; *p* = 0.05; Figure 2).

**FIGURE 3:**
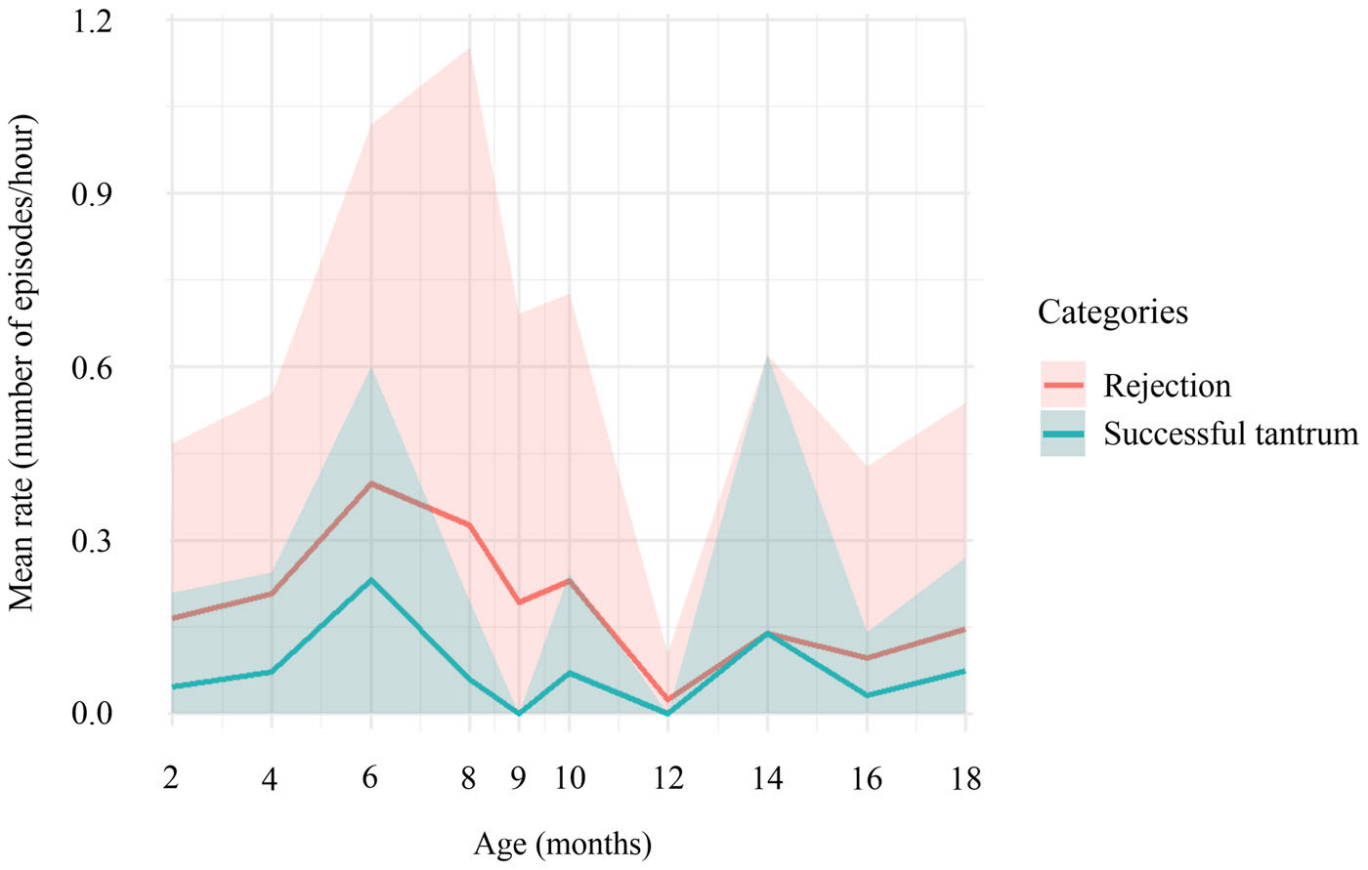
Mean rate (number of episodes per hour) of maternal rejection (red) and tantrum occurrence (green) according to infant age. The shaded area represents the standard deviation. Data are from 12 infants from a wild population of *Sapajus libidinosus* at Fazenda Boa Vista, Brazil.

Maternal rejection was rare (*N* = 47), but its likelihood increased significantly with age (Model 3: estimate ± *SE* = 0.15 ± 0.03; *p* < 0.001). The duration of mild tantrums ranged from 2 to 82 s (mean ± *SD* = 7.54 ± 13.38, *N* = 15), while intense tantrums ranged from 1 to 45 s (mean ± *SD* = 8.19 ± 9.07, *N* = 22). Tantrums only occurred following a maternal rejection, but their occurrence did not vary significantly with age (Model 4: estimate ± *SE* = −0.06 ± 0.10; *p* = 0.53) or infant sex (Model 4: estimate ± *SE* = 0.12 ± 1.38; *p* = 0.93). However, we observed that their occurrence followed a similar pattern to maternal rejections, with most rejections eliciting a tantrum display (*N* = 31; Figure 3). Tantrum absence fully discriminated the outcome of the interaction, meaning that infants never succeed in obtaining maternal care after a rejection if they did not display a tantrum. However, even when displaying tantrums, their chances of success did not increase significantly: of the 31 tantrum displays, infants succeeded 19 times and failed 12. We found no significant effect of tantrum intensity and duration with increased maternal responsiveness (Model 5: mild tantrum estimate ± *SE* = −0.13 ± 0.12; *p* = 0.29; intense tantrum estimate ± *SE* = 0.15 ± 0.13; *p* = 0.22).

To assess whether age‐related patterns during later infancy (i.e., Months 14–18) could obscure effects occurring earlier in development, we repeated Models 4 and 5 considering only the first 12 months of life. The results remained unchanged, with no significant effects of tantrum occurrence and characteristics on maternal responsiveness. These additional analyses are reported in the Supporting Information (Statistical Report S2).

## 4 Discussion

In this study, we investigated whether tantrums effectively influenced maternal investment in wild bearded capuchin monkeys. We predicted an increase in maternal rejection during weaning. Our finding that maternal rejection became more likely as infants aged and weaning began supports our prediction and is consistent with patterns described in several primate species (e.g., *Papio cynocephalus*: Altmann 1980; *Alouatta caraya*: Pavé et al. 2015; *Ateles geoffroyi*: Arbaiza‐Bayona et al. 2022). We further predicted that this rise in maternal rejection would be followed by an increase in tantrum occurrence and that, if tantrums were effective, they would elicit greater maternal responsiveness, as well as a temporary increase in solicitation rates as tantrums were reinforced. This prediction was only partially supported. Although the occurrence of tantrums followed a pattern similar to maternal rejection, we found no significant evidence that tantrums elicited increased maternal responsiveness. Furthermore, neither more intense nor prolonged episodes were associated with greater maternal investment. Moreover, we found no evidence of a temporary increase in infant solicitations for maternal care. Rather, solicitation rates declined significantly with age.

Taken together, these findings suggest a gradual transition toward independence characterized by reciprocal behavioral adjustments within the mother–infant pair. Infants progressively reduce their demands, and mothers become less responsive to attempts at seeking care. In this context, tantrums did not function as an effective strategy for negotiating maternal investment, as proposed by parent–offspring conflict theory (Trivers 1974). One possible explanation for this result is that tantrums are not reinforced by mothers in this population, preventing them from becoming an effective behavioral strategy for obtaining additional care. Instead, as suggested by previous authors, tantrums may reflect the emotional challenges of behavioral regulation during the transition to independence associated with weaning (Altmann 1980; Bateson 1994; Avital and Jablonka 2000). In this sense, tantrums may represent a developmental adjustment through which infants gradually learn to regulate their responses to maternal rejection, rather than a tactic selected for influencing maternal behavior.

Similar results have been reported for rhesus macaques, where maternal responsiveness to infant distress decreased with infant age or the mother's reproductive context (Berman et al. 1993; Devinney et al. 2001). For baboons (*Papio ursinus*), a correlation between tantrum occurrence and environmental conditions was found in two different studies, with differences in maternal responsiveness. Barrett and Henzi (2000) observed that tantrums were rare, but more likely to occur under harsh conditions, and that mothers were responsive to them. Dezeure et al. (2021) found that when infants face weaning during the dry season, they display more tantrums. Mothers increase their investment during the dry season, but they do so regardless of offspring's age, meaning this increase does not seem to happen in response to tantrums. At Fazenda Boa Vista, ecological conditions are relatively stable, with high‐quality food available year‐round (Izar et al. 2012). Given this scenario, and that solicitation rates drop considerably as infants age, mothers might not have to strictly control their energy investment. They can afford to be more tolerant, and infants do not face high costs when care is denied. According to models like Bateson's (1994), when resources are abundant and the environment is safe, mother–infant interactions may be more cooperative than conflict driven. This ecological context may help explain why we see relatively few rejections and why tantrums do not seem to be strongly selected for as a negotiation tool. At a proximate level, in this population, tantrums may be less about influencing the mother, but part of a broader developmental process shaped by the constraints of the environment. Under such conditions, the benefits of escalating solicitation through tantrums may be limited, which could explain why these displays did not increase maternal investment in our dataset.

Social factors may also contribute to the patterns observed. Mother identity appears to strongly influence the relationship between tantrums and infants’ success in obtaining maternal care (Model 5: ICC_motID_ = 0.98; Supporting Information). Due to convergence issues, this variable could not be included as a fixed effect in the statistical model, preventing a more detailed assessment of its specific influence. However, the high ICC suggests that variation in maternal styles may play an important role in shaping these interactions. Infant behavior and development can be influenced by different maternal styles, with research indicating that infants reared by less responsive mothers tend to explore their environment more extensively and engage more frequently in social play (reviewed in Maestripieri 2018; Most and Strum 2020; Omena et al., forthcoming).

In addition to variation in maternal style, access to alternative caregivers may influence infant care‐seeking strategies. Alloparenting is known to play a significant role in the development of infant capuchins (Robinson and O'Brien 1991; Baldovino and Di Bitetti 2008; Fearnside 2023). Although we did not systematically investigate allomaternal care in the present study, our video screening revealed that other group members frequently carried and interacted with infants (but rarely allonurse), particularly during the first 6 months of life. This additional social support may reduce infants’ reliance on maternal investment and help explain why maternal care did not increase following tantrums. Supporting this interpretation, Most and Strum (2020) found that olive baboon (*Papio anubis*) infants with less responsive mothers were more likely to develop secondary attachment figures, such as siblings or other adult group members. Further studies examining infant interaction with nonmaternal group members may therefore provide valuable insights into the broader social dynamics of infant care in capuchin monkeys.

In primates, maternal care is reported to be influenced by offspring sex, with mothers typically favoring the philopatric sex (Maestripieri 2018). In this population, females are philopatric (Izar et al. 2012, Izar et al. 2022) and more sociable and begin engaging in social interactions earlier in their development than males (Omena and Izar 2023). In this study, however, although they solicited more care than males, we found that females had lower success rates in obtaining investment. This could simply reflect a trade‐off between solicitation frequency and maternal responsiveness, or more complex sex‐specific dynamics, such as documented for rhesus macaques, where mothers of males produced milk of higher energy density, whereas mothers of females produced greater quantities of dilute milk (K. Hinde 2009). Future research is necessary to better explore the sex differences observed in our study.

Our findings should also be interpreted in light of several limitations. First, our relatively small sample size limited statistical power and may have reduced our ability to detect subtle effects of tantrum characteristics on maternal responsiveness. Second, although maternal identity appeared to strongly influence the outcomes of infant solicitations, we were unable to formally examine variation in maternal styles. Finally, we did not systematically quantify allomaternal care, which may influence how infants distribute their care‐seeking behaviors across social partners. Future studies incorporating larger samples and detailed measures of maternal style and allocare will be important for clarifying these dynamics.

Overall, our results suggest that tantrums in this population are unlikely to function as an effective strategy for negotiating maternal investment. Instead, they may reflect the challenges infants face while adjusting to decreasing maternal care during the transition to independence (as discussed by Avital and Jablonka 2000). Environmental changes may further shape these dynamics. For instance, recent research has found that habitat anthropization is changing palm fruit availability at Fazenda Boa Vista, one of their main food resources (Izar et al. 2022; Roncero et al. 2023). In harsher environments, where mothers face stronger energetic trade‐offs, patterns of maternal responsiveness and infant behavior may differ. Our study contributes to the body of work on mother–infant co‐regulation and the emergence of behavioral independence in primates. By drawing insights from both human developmental research and comparative behavioral ecology, we gain a richer understanding of how caregiving strategies evolve in response to ecological life‐history constraints.

## Conflicts of Interest

The authors declare no conflicts of interest.

## Supporting information




**Supplementary Material**: dev70157‐sup‐0001‐SuppMat.xlsx

**Supplementary Material**: dev70157‐sup‐0002‐SuppMat.xlsx

**Table S1**. Description of infant and mother behaviors.

**Supplementary Material**: dev70157‐sup‐0004‐Statistical_Report_S1.html

**Supplementary Material**: dev70157‐sup‐0005‐Statistical_Report_S2.html

**Supplementary Material**: dev70157‐sup‐0006‐Video_S1.mp4

## Data Availability

The videos observed in this study are part of a larger database of the Laboratory of Ethology, Development, and Social Interaction at the Institute of Psychology of the University of São Paulo, owned and managed by Patrícia Izar. The videos are also used in other research projects and are available upon reasonable request at *ledsapajus.ip@usp.br*. The data supporting the findings of this study and the R script with the analyses performed are available in the Supporting Information.
