# Supplementary material for: Monkey Meltdowns: Do Tantrums Influence Maternal Investment in Bearded Capuchin Monkeys?
Source: Dev Psychobiol. 2026 May 8;68:e70157. doi: 10.1002/dev.70157 (PMC13155812; doi:10.1002/dev.70157)
Supplement: Supplementary file 3 — Table S1. Description of infant and mother behaviors. [file DEV-68-e70157-s001.pdf]

Supplementary Material: Monkey meltdowns: Do tantrums influence maternal investment in bearded capuchin monkeys?

Behavior	Description
**INFANT**	
Contact solicitation	The infant approaches the mother and looks in her direction. The infant may vocalize and touch the mother.
Suckle solicitation	The infant approaches the mother, looks in her direction and touches her nipples. The infant may vocalize or not.
Suckle	The infant positions their head around the mother's nipples. To be considered a nursing episode, the infant must remain for at least five consecutive seconds in contact with the mother's nipples.
Ride on the back	The infant jumps on or propels themselves onto the mother's back. To be considered a riding episode, the infant must remain for at least five consecutive seconds on the mother's back.
Attempt to suckle	The infant attempts to position their head around the mother's nipples but does not succeed.
Attempt to climb on the back	The infant attempts to jump or propel themselves onto the mother's back but does not succeed.
Approach mother	After the mother's response, the infant moves and decreases the distance between them and the mother.
Move away from mother	After the mother's response, the infant moves and increases the distance between them and the mother.
Vocalize	During a solicitation or after the mother's response, the infant opens their mouth and makes sounds.
Intense distress	After the mother's response, the infant displays behaviors such as pushing, pulling or holding the mother, or throwing themselves on the ground.
Self-scratch	After the mother's response, the infant rubs their body repeatedly with their fingers.
Hold tail	After the mother's response, the infant holds their own tail with their hands. The infant may also place the tail in their mouth.
Allotransportation	After the mother's response, the infant is carried by another individual from the group.

Socialize with another individual	After the mother's response, the infant engages in grooming, face to face interaction or stays in contact with another individual from the group.
Conflict with another individual	After the mother's response, the infant threatens, chases or displays other agonistic behaviors towards another individual from the group.
**MOTHER**	
Move away from infant	After the infant's solicitation or during the interaction, the mother moves and increases the distance between her and the infant.
Approach infant	After the infant's solicitation or during the interaction, the mother moves and decreases the distance between her and the infant.
Turn the body	During the interaction with the infant, the mother changes her body orientation to the opposite side.
Hold the infant	After the infant's solicitation or during the interaction, the mother holds the infant using her hands or feet and stops the infant from touching her.
Remove infant's hand from the nipple	After the infant's solicitation or during the interaction, the mother removes the infant's hand that is holding the nipple.
Hit	After the infant's solicitation or during the interaction, the mother rapidly touches the infant with the palm of her hand.
Bite	After the infant's solicitation or during the interaction, the mother opens and compresses her mouth/teeth on the infant's body.
Push	After the infant's solicitation or during the interaction, the mother touches the infant with her hands or feet and propels them away from her body.
Remove infant from the back	The mother partially turns her back and pushes the infant out of her back, ending the riding episode.
Remove infant from the nipple	The mother pushes the infant away from her nipple, ending the nursing episode.
Self-scratch	After the infant's solicitation, the mother rubs their body repeatedly with their fingers.
Behavior not directed to the infant	After the infant's solicitation or during the interaction, the mother avoids eye contact with the infant and displays behaviors not directed at them, such as foraging, feeding, moving, or socially interacting with individuals other than the infant.

Table S1. Description of infant and mother behaviors.
