# Supplementary material for: Monkey Meltdowns: Do Tantrums Influence Maternal Investment in Bearded Capuchin Monkeys?
Source: Dev Psychobiol. 2026 May 8;68:e70157. doi: 10.1002/dev.70157 (PMC13155812; doi:10.1002/dev.70157)
Supplement: Supplementary file 4 — Supplementary Material: dev70157‐sup‐0004‐Statistical_Report_S1.html [file DEV-68-e70157-s002.html]

Monkey meltdowns: Do tantrums influence maternal investment in bearded capuchin monkeys?


# Monkey meltdowns: Do tantrums influence maternal investment in bearded capuchin monkeys?

# 1 Statistical analyses

This report presents the statistical modeling conducted for the study “*Monkey meltdowns: Do tantrums influence maternal investment in bearded capuchin monkeys?*”. All analyses were performed in R (version 4.5.0).

# 2 Dataset description

The dataset includes a total of 550 episodes of maternal care solicitation from 12 *Sapajus libidinosus* infants. The subjects were filmed weekly from dawn to dusk, from birth to 18 months, using focal animal sampling (Altmann, 1974). To select the episodes analyzed, we screened 5.353 videos in 10 timepoints (every other month from the 2nd to the 18th month, plus the 9th month), totaling 213 hours and 16 minutes of footage. For each episode, we categorized whether the infant succeeded or failed in obtaining maternal care (coded as a binary variable, 0 = failure and 1 = success) and whether the infant exhibited a tantrum (coded as a binary variable, 0 = no tantrum and 1 = with tantrum). When tantrums were present, we categorized them into mild or intense and registered the duration, in seconds, of each tantrum level.

# 3 Adjusting Models

To investigate our hypothesis, we fitted the Generalized Linear Mixed Models (GLMMs) specified below.

## 3.1 Model 1

In this model, our goal was to verify if infant solicitations rate varied according to infant age, sex, and mother identity. Overdispersion was detected and therefore we used a negative binomial distribution. We included the observation time as an offset to control for the different total observation time in each month. Since mother identity did not contribute to the model, we fitted a reduced model including only infant age and sex as fixed effects. Infant identity was included as a random effect.

**Overdispersion test**

```
overdisp(x = rates,
         dependent.position = 8,
         predictor.position = 3)
```

```
    Overdispersion Test - Cameron & Trivedi (1990)

data:  rates
Lambda t test score: = 3.9903, p-value = 0.0001145
alternative hypothesis: Overdispersion
```

**Variable selection and model comparison**

```
# -------------------------------
# Null model (only intercept, offset, and random effect of infID)
# -------------------------------
null_model <- glmer.nb(solicitations.total ~ 1 +
                     offset(log(obsMinutes)) + (1 | infID),
                     data = rates)

# -------------------------------
# Model 1 (adds fixed effects: infant age and sex)
# -------------------------------
model_1<- glmer.nb(solicitations.total ~ age + infSex +
                         offset(log(obsMinutes)) + (1 | infID),
                       data = rates)

# -------------------------------
# Compare null model and Model 1
# -------------------------------
# (tests whether including age + sex improves model fit)
anova(null_model, model_1, test = "Chisq")
```

```
Data: rates
Models:
null_model: solicitations.total ~ 1 + offset(log(obsMinutes)) + (1 | infID)
model_1: solicitations.total ~ age + infSex + offset(log(obsMinutes)) + (1 | infID)
           npar    AIC    BIC  logLik -2*log(L)  Chisq Df Pr(>Chisq)    
null_model    3 570.04 578.40 -282.02    564.04                         
model_1       5 471.90 485.83 -230.95    461.90 102.14  2  < 2.2e-16 ***
---
Signif. codes:  0 '***' 0.001 '**' 0.01 '*' 0.05 '.' 0.1 ' ' 1
```

```
# -------------------------------
# Model 2 (adds mother as an additional fixed effect)
# -------------------------------
model_2 <- glmer.nb(solicitations.total ~ age + infSex + motID +
                         offset(log(obsMinutes)) + (1 | infID),
                       data = rates)

# -------------------------------
# Compare Model 1 and Model 2
# -------------------------------
# (tests whether including mother improves model fit)
anova(model_1, model_2, test = "Chisq")
```

```
Data: rates
Models:
model_1: solicitations.total ~ age + infSex + offset(log(obsMinutes)) + (1 | infID)
model_2: solicitations.total ~ age + infSex + motID + offset(log(obsMinutes)) + (1 | infID)
        npar   AIC    BIC  logLik -2*log(L)  Chisq Df Pr(>Chisq)
model_1    5 471.9 485.83 -230.95     461.9                     
model_2   11 478.3 508.96 -228.15     456.3 5.6017  6     0.4693
```

**Final model**

```
model_1 <- glmer.nb(solicitations.total ~ age + infSex +
                         offset(log(obsMinutes)) + (1 | infID),
                       data = rates)

summ(model_1)
```

|  |  |
| --- | --- |
| Observations | 120 |
| Dependent variable | solicitations.total |
| Type | Mixed effects generalized linear model |
| Family | Negative Binomial(6.6639) |
| Link | log |

|  |  |
| --- | --- |
| AIC | 471.90 |
| BIC | 485.83 |
| Pseudo-R² (fixed effects) | 0.74 |
| Pseudo-R² (total) | 0.82 |

| Fixed Effects | | | | |
| --- | --- | --- | --- | --- |
|  | Est. | S.E. | z val. | p |
| (Intercept) | -1.67 | 0.18 | -9.37 | 0.00 |
| age | -0.18 | 0.02 | -11.89 | 0.00 |
| infSexM | -0.54 | 0.22 | -2.41 | 0.02 |

| Random Effects | | |
| --- | --- | --- |
| Group | Parameter | Std. Dev. |
| infID | (Intercept) | 0.30 |

| Grouping Variables | | |
| --- | --- | --- |
| Group | # groups | ICC |
| infID | 12 | 0.02 |

**See diagnostics**

```
    Shapiro-Wilk normality test

data:  R
W = 0.98397, p-value = 0.1657
```

## 3.2 Model 2

In this model, we fitted a logistic regression model to investigate the effect of age, sex, and mother identity on the probability of infant success in obtaining maternal care. We used the ‘cbind’ function to account for the different proportion of episodes in each month. Since mother identity did not contribute to the model, we fitted a reduced model including only infant age and sex as fixed effects. Infant identity was included as a random effect.

**Variable selection and model comparison**

```
# -------------------------------
# 1. Null model (only intercept + random effect of infID)
# -------------------------------
null_model2 <- glmer(
  cbind(success, failure) ~ 1 + (1 | infID),
  family = binomial(link = "logit"),
  data = dados_proporcoes
)

# -------------------------------
# 2. Model 1 (adds fixed effects: age and sex)
# -------------------------------
model2_1 <- glmer(
  cbind(success, failure) ~ age + infSex + (1 | infID),
  family = binomial(link = "logit"),
  data = dados_proporcoes
)

# Compare null model vs Model 1
# (tests whether including age + infSex improves model fit)
anova(null_model2, model2_1, test = "Chisq")
```

```
Data: dados_proporcoes
Models:
null_model2: cbind(success, failure) ~ 1 + (1 | infID)
model2_1: cbind(success, failure) ~ age + infSex + (1 | infID)
            npar    AIC    BIC  logLik -2*log(L)  Chisq Df Pr(>Chisq)    
null_model2    2 123.66 129.23 -59.828    119.66                         
model2_1       4 110.77 121.92 -51.385    102.77 16.887  2  0.0002154 ***
---
Signif. codes:  0 '***' 0.001 '**' 0.01 '*' 0.05 '.' 0.1 ' ' 1
```

```
# -------------------------------
# 3. Model with mother identity (motID) as fixed effect
# -------------------------------
model2_2 <- glmer(
  cbind(success, failure) ~ age + infSex + (1 | infID) + (1 | motID),
  family = binomial(link = "logit"),
  data = dados_proporcoes
)

# Compare Model 1 vs Model with mother as fixed effect
# (tests whether adding motID improves model fit)
anova(model2_1, model2_2, test = "Chisq")
```

```
Data: dados_proporcoes
Models:
model2_1: cbind(success, failure) ~ age + infSex + (1 | infID)
model2_2: cbind(success, failure) ~ age + infSex + (1 | infID) + (1 | motID)
         npar    AIC    BIC  logLik -2*log(L)  Chisq Df Pr(>Chisq)  
model2_1    4 110.77 121.92 -51.385    102.77                       
model2_2    5 109.82 123.76 -49.910     99.82 2.9502  1    0.08587 .
---
Signif. codes:  0 '***' 0.001 '**' 0.01 '*' 0.05 '.' 0.1 ' ' 1
```

**Final model**

```
model2_1 <- glmer(
  cbind(success, failure) ~ age + infSex + (1 | infID),
  family = binomial(link = "logit"),
  data = dados_proporcoes
)

summ(model2_1)
```

|  |  |
| --- | --- |
| Observations | 120 |
| Dependent variable | cbind(success, failure) |
| Type | Mixed effects generalized linear model |
| Family | binomial |
| Link | logit |

|  |  |
| --- | --- |
| AIC | 110.77 |
| BIC | 121.92 |
| Pseudo-R² (fixed effects) | 0.24 |
| Pseudo-R² (total) | 0.30 |

| Fixed Effects | | | | |
| --- | --- | --- | --- | --- |
|  | Est. | S.E. | z val. | p |
| (Intercept) | 3.75 | 0.51 | 7.31 | 0.00 |
| age | -0.15 | 0.05 | -3.19 | 0.00 |
| infSexM | 1.61 | 0.82 | 1.95 | 0.05 |

| Random Effects | | |
| --- | --- | --- |
| Group | Parameter | Std. Dev. |
| infID | (Intercept) | 0.54 |

| Grouping Variables | | |
| --- | --- | --- |
| Group | # groups | ICC |
| infID | 12 | 0.08 |

**See diagnostics**

## 3.3 Model 3

In this model, we fitted a logistic regression model to investigate the effect of age, sex, and mother identity on the probability of maternal rejection. We used the ‘cbind’ function to account for the different proportion of episodes in each month. Since mother identity did not contribute to the model, we fitted a reduced model including infant age and sex as fixed effects. Infant identity was included as a random effect.

**Variable selection and model comparison**

```
# -------------------------------
# Null model (only intercept and random effect of infID)
# -------------------------------
null_model3 <- glmer(
  cbind(rejection, `no rejection`) ~ 1 + (1 | infID),
  family = binomial(link = "logit"),
  data = dados_proporcoes
)

# -------------------------------
# Model 1 (adds fixed effects: infant age and sex)
# -------------------------------
model3_1 <- glmer(
  cbind(rejection, `no rejection`) ~ age + infSex + (1 | infID),
  family = binomial(link = "logit"),
  data = dados_proporcoes
)

# -------------------------------
# Compare null model and Model 1
# -------------------------------
# (tests whether including age + sex improves model fit compared to the null model)
anova(null_model3, model3_1, test = "Chisq")
```

```
Data: dados_proporcoes
Models:
null_model3: cbind(rejection, `no rejection`) ~ 1 + (1 | infID)
model3_1: cbind(rejection, `no rejection`) ~ age + infSex + (1 | infID)
            npar    AIC    BIC  logLik -2*log(L)  Chisq Df Pr(>Chisq)    
null_model3    2 172.93 178.51 -84.466    168.93                         
model3_1       4 153.95 165.10 -72.974    145.95 22.984  2  1.021e-05 ***
---
Signif. codes:  0 '***' 0.001 '**' 0.01 '*' 0.05 '.' 0.1 ' ' 1
```

```
# -------------------------------
# Model with mother identity (motID) added as a fixed effect
# -------------------------------
model3_mother_fixed <- glmer(
  cbind(rejection, `no rejection`) ~ age + infSex + motID + (1 | infID),
  family = binomial(link = "logit"),
  data = dados_proporcoes
)

# -------------------------------
# Compare Model 1 vs Model with mother as fixed effect
# -------------------------------
# (tests whether including motID improves model fit)
anova(model3_1, model3_mother_fixed, test = "Chisq")
```

```
Data: dados_proporcoes
Models:
model3_1: cbind(rejection, `no rejection`) ~ age + infSex + (1 | infID)
model3_mother_fixed: cbind(rejection, `no rejection`) ~ age + infSex + motID + (1 | infID)
                    npar    AIC    BIC  logLik -2*log(L)  Chisq Df Pr(>Chisq)  
model3_1               4 153.95 165.10 -72.974    145.95                       
model3_mother_fixed   10 154.09 181.96 -67.043    134.09 11.863  6    0.06509 .
---
Signif. codes:  0 '***' 0.001 '**' 0.01 '*' 0.05 '.' 0.1 ' ' 1
```

**Final model**

```
model3_1 <- glmer(
  cbind(rejection, `no rejection`) ~ age + infSex + (1 | infID),
  family = binomial(link = "logit"),
  data = dados_proporcoes
)

summ(model3_1)
```

|  |  |
| --- | --- |
| Observations | 120 |
| Dependent variable | cbind(rejection, `no rejection`) |
| Type | Mixed effects generalized linear model |
| Family | binomial |
| Link | logit |

|  |  |
| --- | --- |
| AIC | 153.95 |
| BIC | 165.10 |
| Pseudo-R² (fixed effects) | 0.16 |
| Pseudo-R² (total) | 0.18 |

| Fixed Effects | | | | |
| --- | --- | --- | --- | --- |
|  | Est. | S.E. | z val. | p |
| (Intercept) | -3.21 | 0.35 | -9.18 | 0.00 |
| age | 0.15 | 0.03 | 4.25 | 0.00 |
| infSexM | -0.73 | 0.45 | -1.63 | 0.10 |

| Random Effects | | |
| --- | --- | --- |
| Group | Parameter | Std. Dev. |
| infID | (Intercept) | 0.27 |

| Grouping Variables | | |
| --- | --- | --- |
| Group | # groups | ICC |
| infID | 12 | 0.02 |

**See diagnostics**

## 3.4 Model 4

In this model, we fitted a logistic regression model to investigate the effect of age, sex, and mother identity on the probability of tantrum occurrence. We used the ‘cbind’ function to account for the different proportion of episodes in each month. Infant identity was included as a random effect.

**Variable selection and model comparison**

```
# -------------------------------
# 1. Null model (only intercept + random effect of infID)
# -------------------------------
null_model4 <- glmer(
  cbind(tantrum, `no tantrum`) ~ 1 + (1 | infID),
  family = binomial(link = "logit"),
  data = dados_proporcoes
)

# -------------------------------
# 2. Model 1 (adds fixed effects: age and sex)
# -------------------------------
model4_1 <- glmer(
  cbind(tantrum, `no tantrum`) ~ age + infSex + (1 | infID),
  family = binomial(link = "logit"),
  data = dados_proporcoes
)

# Compare null model vs Model 1
# (tests whether including age + infSex improves model fit)
anova(null_model4, model4_1, test = "Chisq")
```

```
Data: dados_proporcoes
Models:
null_model4: cbind(tantrum, `no tantrum`) ~ 1 + (1 | infID)
model4_1: cbind(tantrum, `no tantrum`) ~ age + infSex + (1 | infID)
            npar    AIC    BIC  logLik -2*log(L)  Chisq Df Pr(>Chisq)
null_model4    2 51.320 56.895 -23.660    47.320                     
model4_1       4 54.817 65.967 -23.409    46.817 0.5034  2     0.7775
```

```
# -------------------------------
# 3. Model with mother identity (motID) as fixed effect
# -------------------------------
model4_mother_random <- glmer(
  cbind(tantrum, `no tantrum`) ~ age + infSex + (1 | infID) + (1 | motID),
  family = binomial(link = "logit"),
  data = dados_proporcoes
)
# Unlike previous models where motID was included as a fixed effect,
# here we include motID as a random intercept because it has many levels
# and including it as a fixed effect causes convergence issues.

# -------------------------------
# Compare Model 1 vs Model with mother as fixed effect
# -------------------------------
# (tests whether adding motID improves model fit)
anova(model4_1, model4_mother_random, test = "Chisq")
```

```
Data: dados_proporcoes
Models:
model4_1: cbind(tantrum, `no tantrum`) ~ age + infSex + (1 | infID)
model4_mother_random: cbind(tantrum, `no tantrum`) ~ age + infSex + (1 | infID) + (1 | motID)
                     npar    AIC    BIC  logLik -2*log(L) Chisq Df Pr(>Chisq)
model4_1                4 54.817 65.967 -23.409    46.817                    
model4_mother_random    5 56.817 70.755 -23.409    46.817     0  1          1
```

**Final model**

```
model4_1 <- glmer(
  cbind(tantrum, `no tantrum`) ~ age + infSex + (1 | infID),
  family = binomial(link = "logit"),
  data = dados_proporcoes
)

summ(model4_1)
```

|  |  |
| --- | --- |
| Observations | 120 |
| Dependent variable | cbind(tantrum, `no tantrum`) |
| Type | Mixed effects generalized linear model |
| Family | binomial |
| Link | logit |

|  |  |
| --- | --- |
| AIC | 54.82 |
| BIC | 65.97 |
| Pseudo-R² (fixed effects) | 0.02 |
| Pseudo-R² (total) | 0.29 |

| Fixed Effects | | | | |
| --- | --- | --- | --- | --- |
|  | Est. | S.E. | z val. | p |
| (Intercept) | 1.31 | 1.10 | 1.18 | 0.24 |
| age | -0.06 | 0.10 | -0.63 | 0.53 |
| infSexM | 0.12 | 1.38 | 0.09 | 0.93 |

| Random Effects | | |
| --- | --- | --- |
| Group | Parameter | Std. Dev. |
| infID | (Intercept) | 1.12 |

| Grouping Variables | | |
| --- | --- | --- |
| Group | # groups | ICC |
| infID | 12 | 0.28 |

**See diagnostics**

## 3.5 Model 5

In this model, we fitted a logistic regression model to investigate the effect of mild and intense tantrums durations, infant sex, and mother identity on the probability of infant success in obtaining maternal care. Infant identity was included as a random effect.

**Variable selection and model comparison**

```
# -------------------------------
# 1. Null model (only intercept + random effect of infID)
# -------------------------------
null_model5 <- glmer(
  infSuccess ~ 1 + (1 | infID),
  family = binomial(link = "logit"),
  data = eventos_birra
)

# -------------------------------
# 2. Model 5.1: fixed effects mTantrum and iTantrum
# -------------------------------
model5_1 <- glmer(
  infSuccess ~ mTantrum + iTantrum + (1 | infID),
  family = binomial(link = "logit"),
  data = eventos_birra
)

# Compare null model vs Model 5.1
anova(null_model5, model5_1, test = "Chisq")
```

```
Data: eventos_birra
Models:
null_model5: infSuccess ~ 1 + (1 | infID)
model5_1: infSuccess ~ mTantrum + iTantrum + (1 | infID)
            npar    AIC    BIC  logLik -2*log(L)  Chisq Df Pr(>Chisq)
null_model5    2 37.567 40.435 -16.784    33.567                     
model5_1       4 41.167 46.903 -16.583    33.167 0.4009  2     0.8184
```

```
# -------------------------------
# 3. Model 5.2: add mother (motID) as a fixed effect
# -------------------------------
# Note: Unlike previous models where motID was included as a fixed effect,
# here we include motID as a random intercept because it has many levels
# and including it as a fixed effect causes convergence issues.
model5_2 <- glmer(
  infSuccess ~ mTantrum + iTantrum + (1 | motID) + (1 | infID),
  family = binomial(link = "logit"),
  data = eventos_birra
)

# Compare Model 5.1 vs Model 5.2
anova(model5_1, model5_2, test = "Chisq")
```

```
Data: eventos_birra
Models:
model5_1: infSuccess ~ mTantrum + iTantrum + (1 | infID)
model5_2: infSuccess ~ mTantrum + iTantrum + (1 | motID) + (1 | infID)
         npar    AIC    BIC  logLik -2*log(L)  Chisq Df Pr(>Chisq)   
model5_1    4 41.167 46.903 -16.583    33.167                        
model5_2    5 35.814 42.984 -12.907    25.814 7.3525  1   0.006697 **
---
Signif. codes:  0 '***' 0.001 '**' 0.01 '*' 0.05 '.' 0.1 ' ' 1
```

```
# Compare Null Model vs Model 5.2
anova(null_model5, model5_2, test = "Chisq")
```

```
Data: eventos_birra
Models:
null_model5: infSuccess ~ 1 + (1 | infID)
model5_2: infSuccess ~ mTantrum + iTantrum + (1 | motID) + (1 | infID)
            npar    AIC    BIC  logLik -2*log(L)  Chisq Df Pr(>Chisq)  
null_model5    2 37.567 40.435 -16.784    33.567                       
model5_2       5 35.814 42.984 -12.907    25.814 7.7534  3    0.05139 .
---
Signif. codes:  0 '***' 0.001 '**' 0.01 '*' 0.05 '.' 0.1 ' ' 1
```

**Final model**

```
model5_2 <- glmer(
  infSuccess ~ mTantrum + iTantrum + (1 | motID) + (1 | infID),
  family = binomial(link = "logit"),
  data = eventos_birra
)

summ(model5_2)
```

|  |  |
| --- | --- |
| Observations | 31 |
| Dependent variable | infSuccess |
| Type | Mixed effects generalized linear model |
| Family | binomial |
| Link | logit |

|  |  |
| --- | --- |
| AIC | 35.81 |
| BIC | 42.98 |
| Pseudo-R² (fixed effects) | 0.02 |
| Pseudo-R² (total) | 0.98 |

| Fixed Effects | | | | |
| --- | --- | --- | --- | --- |
|  | Est. | S.E. | z val. | p |
| (Intercept) | 9.91 | 6.54 | 1.51 | 0.13 |
| mTantrum | -0.13 | 0.12 | -1.06 | 0.29 |
| iTantrum | 0.15 | 0.13 | 1.22 | 0.22 |

| Random Effects | | |
| --- | --- | --- |
| Group | Parameter | Std. Dev. |
| infID | (Intercept) | 0.00 |
| motID | (Intercept) | 11.52 |

| Grouping Variables | | |
| --- | --- | --- |
| Group | # groups | ICC |
| infID | 9 | 0.00 |
| motID | 6 | 0.98 |

**See diagnostics**
