# Supplementary material for: Monkey Meltdowns: Do Tantrums Influence Maternal Investment in Bearded Capuchin Monkeys?
Source: Dev Psychobiol. 2026 May 8;68:e70157. doi: 10.1002/dev.70157 (PMC13155812; doi:10.1002/dev.70157)
Supplement: Supplementary file 5 — Supplementary Material: dev70157‐sup‐0005‐Statistical_Report_S2.html [file DEV-68-e70157-s003.html]

Monkey meltdowns: Do tantrums influence maternal investment in bearded capuchin monkeys?


# Monkey meltdowns: Do tantrums influence maternal investment in bearded capuchin monkeys?

##

## Model 4

```
# -------------------------------
# 1. Null model (only intercept + random effect of infID)
# -------------------------------
null_model4 <- glmer(
  cbind(tantrum, `no tantrum`) ~ 1 + (1 | infID),
  family = binomial(link = "logit"),
  data = dados_proporcoes
)

# -------------------------------
# 2. Model 1 (adds fixed effects: age and sex)
# -------------------------------
model4_1 <- glmer(
  cbind(tantrum, `no tantrum`) ~ age + infSex + (1 | infID),
  family = binomial(link = "logit"),
  data = dados_proporcoes
)

# Compare null model vs Model 1
# (tests whether including age + infSex improves model fit)
anova(null_model4, model4_1, test = "Chisq")
```

```
Data: dados_proporcoes
Models:
null_model4: cbind(tantrum, `no tantrum`) ~ 1 + (1 | infID)
model4_1: cbind(tantrum, `no tantrum`) ~ age + infSex + (1 | infID)
            npar    AIC    BIC  logLik -2*log(L)  Chisq Df Pr(>Chisq)
null_model4    2 40.700 45.562 -18.350    36.700                     
model4_1       4 43.703 53.426 -17.852    35.703 0.9972  2     0.6074
```

```
# -------------------------------
# 3. Model with mother identity (motID) as fixed effect
# -------------------------------
model4_mother_random <- glmer(
  cbind(tantrum, `no tantrum`) ~ age + infSex + (1 | infID) + (1 | motID),
  family = binomial(link = "logit"),
  data = dados_proporcoes
)
# Unlike previous models where motID was included as a fixed effect,
# here we include motID as a random intercept because it has many levels
# and including it as a fixed effect causes convergence issues.

# -------------------------------
# Compare Model 1 vs Model with mother as fixed effect
# -------------------------------
# (tests whether adding motID improves model fit)
anova(model4_1, model4_mother_random, test = "Chisq")
```

```
Data: dados_proporcoes
Models:
model4_1: cbind(tantrum, `no tantrum`) ~ age + infSex + (1 | infID)
model4_mother_random: cbind(tantrum, `no tantrum`) ~ age + infSex + (1 | infID) + (1 | motID)
                     npar    AIC    BIC  logLik -2*log(L) Chisq Df Pr(>Chisq)
model4_1                4 43.703 53.426 -17.852    35.703                    
model4_mother_random    5 45.703 57.857 -17.852    35.703     0  1     0.9999
```

**Final model**

```
model4_1 <- glmer(
  cbind(tantrum, `no tantrum`) ~ age + infSex + (1 | infID),
  family = binomial(link = "logit"),
  data = dados_proporcoes
)

summ(model4_1)
```

|  |  |
| --- | --- |
| Observations | 84 |
| Dependent variable | cbind(tantrum, `no tantrum`) |
| Type | Mixed effects generalized linear model |
| Family | binomial |
| Link | logit |

|  |  |
| --- | --- |
| AIC | 43.70 |
| BIC | 53.43 |
| Pseudo-R² (fixed effects) | 0.04 |
| Pseudo-R² (total) | 0.53 |

| Fixed Effects | | | | |
| --- | --- | --- | --- | --- |
|  | Est. | S.E. | z val. | p |
| (Intercept) | 0.65 | 1.69 | 0.39 | 0.70 |
| age | 0.16 | 0.16 | 0.98 | 0.33 |
| infSexmale | -0.07 | 1.94 | -0.04 | 0.97 |

| Random Effects | | |
| --- | --- | --- |
| Group | Parameter | Std. Dev. |
| infID | (Intercept) | 1.85 |

| Grouping Variables | | |
| --- | --- | --- |
| Group | # groups | ICC |
| infID | 12 | 0.51 |

**See diagnostics**

## Model 5

**Variable selection and model comparison**

```
# -------------------------------
# 1. Null model (only intercept + random effect of infID)
# -------------------------------
null_model5 <- glmer(
  infSuccess ~ 1 + (1 | infID),
  family = binomial(link = "logit"),
  data = eventos_birra
)

# -------------------------------
# 2. Model 5.1: fixed effects mTantrum and iTantrum
# -------------------------------
model5_1 <- glmer(
  infSuccess ~ mTantrum + iTantrum + (1 | infID),
  family = binomial(link = "logit"),
  data = eventos_birra
)

# Compare null model vs Model 5.1
anova(null_model5, model5_1, test = "Chisq")
```

```
Data: eventos_birra
Models:
null_model5: infSuccess ~ 1 + (1 | infID)
model5_1: infSuccess ~ mTantrum + iTantrum + (1 | infID)
            npar    AIC    BIC  logLik -2*log(L)  Chisq Df Pr(>Chisq)
null_model5    2 31.586 34.178 -13.793    27.586                     
model5_1       4 35.173 40.357 -13.587    27.173 0.4128  2     0.8135
```

```
# -------------------------------
# 3. Model 5.2: add mother (motID) as a fixed effect
# -------------------------------
# Note: Unlike previous models where motID was included as a fixed effect,
# here we include motID as a random intercept because it has many levels
# and including it as a fixed effect causes convergence issues.
model5_2 <- glmer(
  infSuccess ~ mTantrum + iTantrum + (1 | motID) + (1 | infID),
  family = binomial(link = "logit"),
  data = eventos_birra
)

# Compare Model 5.1 vs Model 5.2
anova(model5_1, model5_2, test = "Chisq")
```

```
Data: eventos_birra
Models:
model5_1: infSuccess ~ mTantrum + iTantrum + (1 | infID)
model5_2: infSuccess ~ mTantrum + iTantrum + (1 | motID) + (1 | infID)
         npar    AIC    BIC   logLik -2*log(L)  Chisq Df Pr(>Chisq)   
model5_1    4 35.173 40.357 -13.5867    27.173                        
model5_2    5 28.783 35.262  -9.3915    18.783 8.3904  1   0.003772 **
---
Signif. codes:  0 '***' 0.001 '**' 0.01 '*' 0.05 '.' 0.1 ' ' 1
```

```
# Compare Null Model vs Model 5.2
anova(null_model5, model5_2, test = "Chisq")
```

```
Data: eventos_birra
Models:
null_model5: infSuccess ~ 1 + (1 | infID)
model5_2: infSuccess ~ mTantrum + iTantrum + (1 | motID) + (1 | infID)
            npar    AIC    BIC   logLik -2*log(L)  Chisq Df Pr(>Chisq)  
null_model5    2 31.586 34.178 -13.7931    27.586                       
model5_2       5 28.783 35.262  -9.3915    18.783 8.8032  3    0.03202 *
---
Signif. codes:  0 '***' 0.001 '**' 0.01 '*' 0.05 '.' 0.1 ' ' 1
```

**Final model**

```
model5_2 <- glmer(
  infSuccess ~ mTantrum + iTantrum + (1 | motID) + (1 | infID),
  family = binomial(link = "logit"),
  data = eventos_birra
)

summ(model5_2)
```

|  |  |
| --- | --- |
| Observations | 27 |
| Dependent variable | infSuccess |
| Type | Mixed effects generalized linear model |
| Family | binomial |
| Link | logit |

|  |  |
| --- | --- |
| AIC | 28.78 |
| BIC | 35.26 |
| Pseudo-R² (fixed effects) | 0.01 |
| Pseudo-R² (total) | 0.99 |

| Fixed Effects | | | | |
| --- | --- | --- | --- | --- |
|  | Est. | S.E. | z val. | p |
| (Intercept) | 10.71 | 6.10 | 1.76 | 0.08 |
| mTantrum | -0.12 | 0.12 | -1.00 | 0.32 |
| iTantrum | 0.18 | 0.16 | 1.13 | 0.26 |

| Random Effects | | |
| --- | --- | --- |
| Group | Parameter | Std. Dev. |
| infID | (Intercept) | 0.00 |
| motID | (Intercept) | 19.09 |

| Grouping Variables | | |
| --- | --- | --- |
| Group | # groups | ICC |
| infID | 8 | 0.00 |
| motID | 5 | 0.99 |

**See diagnostics**
